# Distinct Nutrient Intake Style in Inhabitants of Ultra-High-Altitude Areas in North of Tibet, China: A Cross-Sectional Study Based on Newly Developed Tibetan Food Frequency Questionnaires

**DOI:** 10.3389/fnut.2021.743896

**Published:** 2021-12-23

**Authors:** Zhen Xiao, Xianzhi Sun, Duoji Zhaxi, Fan Zhang, Yuchen Ji, Tingting Cheng, Xiaofeng Li, Xiaoguang Xu

**Affiliations:** ^1^Institute of High Altitude Medicine, People's Hospital of Naqu Affiliated to Dalian Medical University, Naqu, China; ^2^Department of Obstetrics and Gynecology, First Affiliated Hospital of Dalian Medical University, Dalian, China; ^3^Department of Epidemiology and Health Statistics, Dalian Medical University, Dalian, China; ^4^Department of Neurosurgery, Second Affiliated Hospital of Dalian Medical University, Dalian, China

**Keywords:** nutrition intake, food frequency questionnaire, high altitude, Tibet, fat, sodium, vitamin C

## Abstract

Dietary pattern is quite distinct among the inhabitants of high-altitude areas because of environmental and geographical uniqueness; hence, it is important to investigate this data as accurately as possible. However, very few data are related to these populations up to now. Based on the food frequency questionnaire (FFQ) used in the Chinese population, a revised Tibetan edition was developed with respect to the lifestyle in high-altitude areas. After assessment of validity and reproducibility, a nutrition intake survey was conducted among 1,071 randomly sampled Tibetan people. In addition, the Bland–Altman approach was used to compare the agreement between the two dietary tools. For the reproducibility analysis, intraclass correlation coefficients (ICC) were calculated to examine the agreement of food groups and nutrients from the two FFQs (FFQ1 and FFQ2). Nutrient intake was calculated using food composition tables. For the validity analysis, Pearson's correlation of food groups intakes varied from 0.22 to 0.91 (unadjusted). The correlations of nutrients ranged from 0.24 to 0.76 (unadjusted). In the analysis of reliability, the ICC of food groups varied from 0.27 to 0.70 (unadjusted). The ICC of nutrient intakes ranged from 0.22 to 0.87 (unadjusted). The results of nutritional analysis showed that ~25% of foods consumed frequently were traditional Tibetan foods. However, traditional Han foods were frequently consumed. In addition, the energy, iron, and protein intakes for male or female subjects were close to the Chinese Dietary Nutrient Reference Intake (Chinese DRIs); however, fat and sodium intakes were significantly higher than the Chinese DRIs. Interestingly, lower intakes of other types of nutrition, such as vitamin C were detected in people living in high-altitude areas. Our data indicated that excess consumption of fat and sodium and insufficient intake of vitamin C were common among Tibetan people, as compared with the most Chinese people living in the plateau areas. More investigations are needed to reveal the association between the food intake style and high-altitude endemic diseases.

## Introduction

Diet plays an important role in non-communicable diseases, such as type 2 diabetes mellitus, cardiovascular disease, and certain types of cancers. In recent years, many countries including China have published their national dietary guidelines based on the health and nutrition survey data (1–3). However, the national survey in China was elaborated and conducted on a large scale, people living in high-altitude areas, such as Tibet and who have distinct dietary preferences were not specifically investigated and discussed. Naqu City, north of Tibet, has an area of 43 km^2^ and a population of 500,000, with an average altitude of 4,000 m. It is thought that this special geographical and climatic (hypoxic and hypobaric) environment distinguishes this area from other parts of the country. Moreover, most people living in Naqu herded cattle for a living. Because of the unique environmental features, the local residents have a relatively unique life style (4). It is valuable to investigate dietary intakes and assess the nutritional status of inhabitants.

To explore the diet-disease association, an open prospective cohort study, the China Health and Nutrition Survey (CHNS), has been conducted across nine provinces in China (5). The CHNS is a currently ongoing data collection of the University of North Carolina-Chapel Hill and the Chinese Center for Disease Control and Prevention (CCDC). In these dietary intakes surveys, several types of instruments are used, such as the 3-day 24-h diet recall record (3R24), food intake record (FR), and the food frequency questionnaire (FFQ) (6). The 3R24 and FR usually evaluate dietary intakes temporarily. Time, literacy, and economic constraints hinder their application in large-scale population studies (7). However, the FFQ confers relatively low burden and is inexpensive. Furthermore, it can estimate the nutritional status over a relatively long time, ranging from weeks to years (7). So the FFQ has been frequently used in nutritional epidemiology studies (8, 9). At present, although different language revisions of FFQs are available (10, 11), a Tibetan version is still lacking. It is therefore important to develop a specific Tibetan FFQ. Food intake largely varies based on ethnicity, social, and cultural backgrounds of the study population. Hence, dietary assessment methods must be evaluated for validity and reproducibility to improve the application and accuracy within specific population samples (12) and reduce bias levels related to disease occurrence (13).

Naqu is bordered by Qinghai and Sichuan. With consummation of the Qinghai-Tibet Railway, more food and daily necessities are transported to Tibet from Sichuan, Qinghai, and other inland areas. The dietary pattern of inhabitants is under the influence of inland areas. Therefore, we decided to compare nutrition status of inhabitants in Naqu with CHNS data. A number of studies have evaluated the nutritional status of this specific population by analyzing data from the CHNS as well (14, 15).

Hence, our study aimed to design a specific semi-quantitative FFQ of Tibetan inhabitants, assess the validity and reproducibility of FFQ, and evaluate the status of diet and nutrition.

## Materials and Methods

### Survey Design, Sample Size, and Participants

Tibet is known as the “Third Pole” and located on the highest plateau of the world, with 86% of the region at an altitude of at least 4,000 m. The economical activities of most Tibetans are farming and animal husbandry mainly (16). Naqu is a prefecture-level city in the north of the Chinese autonomous region of Tibet. The average altitude is more than 4,500 m above sea level. Naqu contains 89 townships, 25 towns, and 1,283 villages. Specifically, the city includes Anduo, Nierong, and Shenza, while others counties are rural areas.

A cross-sectional survey was designed to evaluate the nutritional status of inhabitants in Naqu. Participants were recruited from the city and the rural area using simple random sampling. The study was approved by the Ethics Review Committee, Naqu People's Hospital. All participants signed a consent form before completing the interview.

The purpose of our study was to measure the dietary intakes of inhabitants of high-altitude areas. The sample size equations were as follows $n={\left(\frac{t_{\frac{\alpha }{2}}}{\delta }\right)}^{2}p\left(1-p\right)$. The proportion (*p* = 0.5) was set to generate the largest sample size. The desired margin of error (δ) was equal to 3%. The survey result will be closer to the real situation. In statistics, the α value is generally 0.05. In other words, the probability of a type I error was set at 0.05. By using this sample size equations, we can estimate 95% proportion with sufficient precision. The calculated sample size was 1,067, which was nutrition survey for Naqu population.

**Figure 1:**
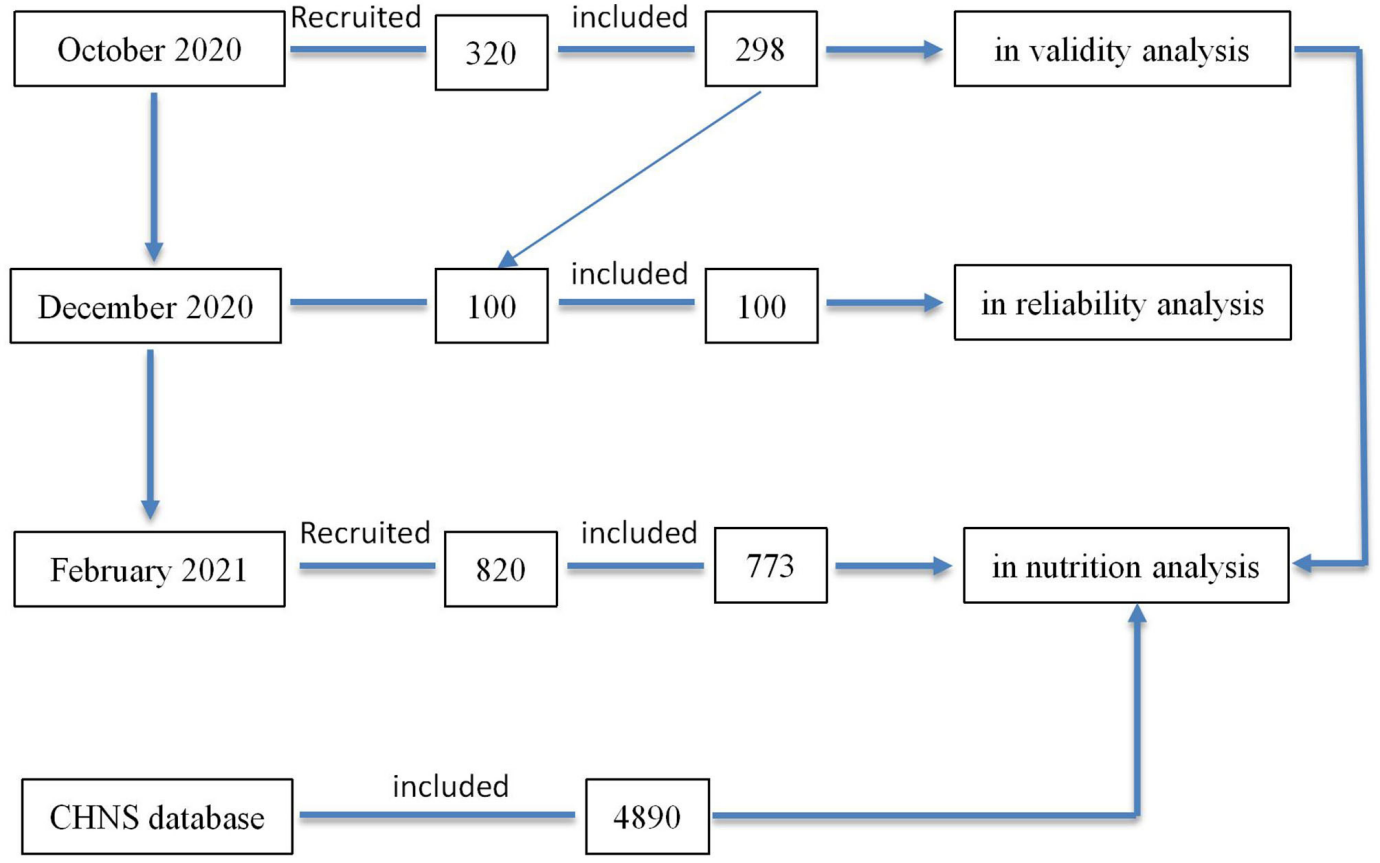
The number of participants in different surveys and survey process.

**Figure 2:**
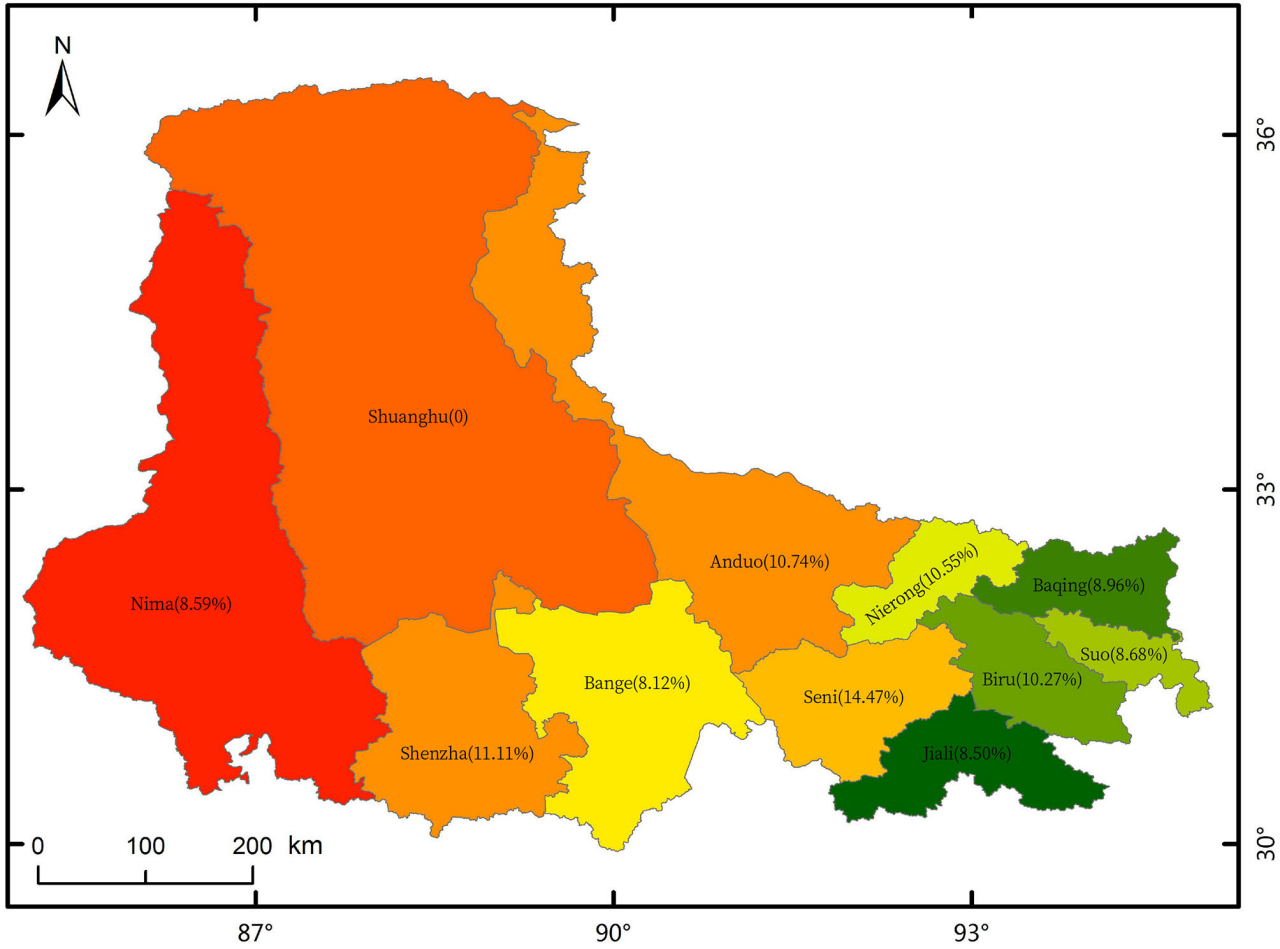
Percentages of participants from 10 different counties.

Before initiating the nutritional status of Tibetan people, a suitable FFQ was designed, and the 320 participants were recruited from Naqu town and rural areas to test the validity of this new-designed questionnaire. In actual, a total of 298 participants completed the questionnaires (93.13%). After this validity analysis, 2 months later, we re-investigated 100 people (out of 298 participants) to complete the self-developed FFQ again for reproducibility analysis. All of them completed the questionnaires. After confirming the validity and reproducibility, another 820 people were investigated for assessing dietary intakes. These interviewees were sampled from Naqu town and rural areas. In total, 773 participants of the 820 interviewees finished their tasks. Finally, 1,071 participants (298 add to 773) were included in dietary intakes analysis (as shown in Figure 1). The percentages of participants from 10 different counties are depicted as Figure 2. Meanwhile, according to the data from the 2011 CHNS (17), 4,890 participants from six low-latitude provinces were extracted from CHNS database (as shown in Figure 1).

Enrolled participants in the study were local inhabitants living in Tibet Naqu for >5 years. Inhabitants aged between 18 and 65 years who independently completed the questionnaires were included. However, pregnant participants with clinical illnesses or nutritional diseases were excluded. The written informed consent was obtained from all subjects. All participants were interviewed face-to-face by trained professional interviewers.

### Questionnaire Development and Data Collection

We developed a new specific FFQ for Tibetan population. Based on an FFQ used in the Chinese population (18), a new FFQ was designed in Tibetan by three Tibetan members who could communicate in both native Tibetan language and Mandarin. The new Tibetan version FFQ included some Tibetan traditional foods, such as Zanba (a kind of flour prepared after grinding frying Highland barley), Ginseng fruit (a local fern in north of Qinghai-Tibet highland area), Tibetan milk tea (made with milk and sugar), and Tibetan salt cream tea (a beverage made of salt, tea, and oil refined from milk). The Tibetan FFQ was divided into two parts. The first part was specific to the study population and was composed of two sections about the basic characteristics of the participants and the physical conditions. The second part included 125 food items, with three sections: the food list, average intake, and frequency of intake. It quantified consumption by frequency, and the frequencies of consumption were divided into daily, weekly, monthly, yearly, and never. The quantitative variables were measured in gram (g).

In addition, the 3R24 used in the Chinese population (18) was translated to Tibetan version. Tibetan version 3R24 can record all foods and beverages consumed by the participants on 3 consecutive days which should include 2 working days and 1 weekend. For each 3R24, besides the consumed food portion size, the research obtained information about the time when the meal was consumed, the place where the meal was consumed, the actual food consumed, and the preparation method. The mean 3R24 data were used as the standard to measure the validity of the FFQ.

We finished data collection of FFQ and 3R24 according to a working manual. The working manual, as the standard tool for all the researchers in this project, included the protocol, questionnaire, explanations for each question, and food list. Systematic training was provided on three occasions before commencing data collection. First, the trained researchers used the Tibetan FFQ to ask 320 participants about eating frequency and portion size of 125 food items in October 2020 (named FFQ1). Second, dietary intakes data of 100 participants were collected two times in December 2020 (named FFQ2) over a period of 2 months. Finally, the researchers used the FFQ that was evaluated for validity and reproducibility to collect the dietary intakes data of 820 participants in February 2021. To reduce error, the researchers used a food model where in the participants estimated the quantity of foods consumed. In addition, we collected one 3R24 questionnaire data when the FFQ1 was finished. A food list was used to remind the subjects of forgotten foods. The data of one participant for whom the mean 3R24 dietary recall indicated an implausible energy intake (<450 kcal or >5,000 kcal) were excluded (19). The consumption information of several specific food items, such as salt, soy source, and cooking oil were collected on the basics of the whole family and then averaged for the individual.

All the interviewers were trained to standardize administration of the questionnaires at least 1 week prior commencing the surveys. At least two members were Tibetan and could communicate in both the native Tibetan language and Mandarin. During the survey, a data checking system was employed, which involved all interviewers checking their own data with another interviewer. Subjects were re-interviewed if wrong answers to key questions or missing values were identified.

### Dietary Intake Assessment and Nutrient Reference Values

We compared the dietary intakes of Tibetan inhabitants with the nutrient reference values. The “Chinese Dietary Nutrient Reference Intakes (Chinese DRIs)” are a set of reference values for the average daily nutrient intakes set to ensure reasonable nutrient intake. DRIs include four parameters: estimated average requirement (EAR), recommended nutrient intake (RNI), adequate intake (AI), and tolerable upper intake level (UL). RNI is age and gender specific and is set to meet the needs of 97–98% of the people in a group. AI is set at a level that is thought to meet the needs for 100% of the people in a given group. RNI and AI are goals established by the Chinese Nutrition Society for nutrient intake in individuals (20). However, for some nutrients, there are not enough data available to establish an RNI, so the AI target value is used (21). As dietary recommendations are different for male and female subjects, we compared daily nutrient intakes of inhabitants with the RNI or AI from the Chinese DRIs (17), based on sex.

The CHNS is a currently ongoing data collection of the University of North Carolina-Chapel Hill and the Chinese Center for Disease Control and Prevention (CCDC). This publicly available longitudinal data apply a multistage and random cluster procedure for data collection across nine provinces. The CHNS was initiated in 1989 and has been followed-up every 2–4 years with a focus on assessing the relationships between the sociological, and demographic transformation and the health and nutritional status (5). Further information can be found from the CHNS official website (http://www.cpc.unc.edu/projects/china) or in the paper published by Zhang et al. (22). We applied only the individual level dataset for this research (2011CHNS).

### Statistical Analysis

All data were entered two times into the Epidata 3.1 software.

First, the characteristics of the participants were described using frequencies with percentages (%) and means with SD for categorical and continuous data, respectively. Second, the food items from questionnaires were grouped according to the food component. All individual nutrient intakes were computed based on the Ingredient List of Chinese Food 2018 version 6 (23) using R 3.6.1 software. As for Tibetan food items, we referred to the similar nutrient content or category food to calculated food nutritional values. For example, we calculated nutritional values of Zanba, by using nutritional values of flour. Food groups and nutrient intakes were log-transformed to improve normality. Then, energy-adjusted food groups and nutrient intakes were computed using the residual method (24). Third, to access the validity of the FFQ, the Pearson's correlation coefficients of FFQ1 and 3R24 were calculated. In addition, the Bland–Altman approach (25) was used to visually compare the consistency between the two dietary tools. The plots included lines for the mean difference and the limits of agreement (LOA), defined as the mean difference ± 1.96 × SD. For the reproducibility analysis, intraclass correlation coefficients (ICC) were calculated to examine the agreement in food groups and nutrients from the two FFQs (FFQ1 and FFQ2), respectively. Finally, for the dietary intakes analysis, we used the Shapiro–Wilk test to check whether nutrient intakes had a normal distribution. Because the data for nutrient intakes had a non-normal distribution, we compared the differences with nutrient reference values using the median values. To evaluate the nutritional status of Naqu inhabitants, we compared dietary intakes with Chinese DRIs using one-sample Wilcoxon signed rank. We used the Wilcoxon rank sum test to determine whether our data were significantly different from the 2011CHNS values as well.

All the analyses were performed using the Statistical Package for the Social Science (SPSS, IBM, NY, USA) software, version 26.0. For all analyses, *p* < 0.05 was considered to indicate statistically significant differences.

## Results

### Validity and Reproducibility of FFQ

Among the initial 320 participants, 22 individuals were excluded, because of clinical illnesses (*n* = 6), nutritional disease (*n* = 4), and younger than 18 years old (*n* = 12). Thus, 298 (93.13%) participants completed both the 3R24 and FFQ1 in validation analysis, with a mean age of 32.2 years. More than 50% participants had normal BMI (kg/m^2^) (underweight: <18.5, normal: 18.5–24.9, over weight: 25–29.9, and obese: ≥30) based on the international classification for BMI (26). The majority of participants had low education, either primary school-level educated or illiterate. About 60% participants were herdsmen either in city or rural. Surprisingly, over 80% of the overall study population never smoked or consumed alcohol. The characteristics of study population are described in Supplementary Table 1.

Table 1 reports the Pearson's correlation coefficients in 11 food groups and 16 nutrients intakes between FFQ1 and 3R24. The Pearson's correlation values (unadjusted) of food groups and nutrient intakes were statistically significant. The ICC of 11 food groups and 16 nutrients are reported in Table 1. The ICC (unadjusted) of food groups and nutrient intakes were statistically significant as well.

**Table 1:** The correlation coefficients of validation and reliability analysis.

	**r values of validation**		**r values of reliability**	
	**Unadjusted**	**Energy-adjusted**	**Unadjusted**	**Energy-adjusted**
**Food Groups**				
Grains	0.51^**^	0.32^**^	0.43^**^	0.34^**^
Meat	0.56^**^	0.61^**^	0.68^**^	0.68^**^
Egg	0.25^**^	0.26^**^	0.69^**^	0.48^**^
Beans	0.74^**^	0.67^**^	0.61^**^	0.51^**^
Milk	0.33^**^	0.33^**^	0.39^**^	0.49^**^
Sweet	0.28^**^	0.24^**^	0.37^**^	0.24^*^
Fresh Vegetable	0.78^**^	0.72^**^	0.61^**^	0.59^**^
Fresh Fruits	0.22^**^	0.23^**^	0.50^**^	0.47^**^
Tea and beverages	0.23^**^	0.21^**^	0.70^**^	0.59^**^
Vegetable Oil	0.71^**^	0.99^**^	0.27^*^	0.25^**^
Salt	0.91^**^	0.84^**^	0.49^**^	0.49^**^
**Nutrient**				
Energy (kcal/d)	0.37^**^		0.68^**^	
Protein (g/d)	0.27^**^	0.43^**^	0.29^**^	0.34^**^
Fat (g/d)	0.48^**^	0.30^**^	0.22^*^	0.35^**^
Carbohydrate (g/d)	0.38^**^	0.29^**^	0.26^*^	0.22^**^
Dietary fiber (g/d)	0.30^**^	0.23^**^	0.64^**^	0.31^**^
Cholesterol (mg/d)	0.30^**^	0.25^**^	0.47^**^	0.58^**^
Vitamin B1 (mg/d)	0.24^**^	0.24^**^	0.36^**^	0.36^**^
Vitamin B3 (mg/d)	0.34^**^	0.35^**^	0.41^**^	0.41^**^
Vitamin C (mg/d)	0.75^**^	0.75^**^	0.66^**^	0.65^**^
Potassium (mg/d)	0.30^**^	0.41^**^	0.87^**^	0.82^**^
Magnesium (mg/d)	0.54^**^	0.59^**^	0.52^**^	0.84^**^
Zinc (mg/d)	0.41^**^	0.39^**^	0.35^**^	0.36^**^
Sodium (mg/d)	0.76^**^	0.61^**^	0.48^**^	0.48^**^
Iron (mg/d)	0.29^**^	0.23^**^	0.29^**^	0.35^**^
Manganese (mg/d)	0.51^**^	0.25^**^	0.50^**^	0.51^**^
Selenium (ug/d)	0.41^**^	0.34^**^	0.35^**^	0.40^**^

*r, correlation coefficient*,

* *p < 0.05*,

** *p < 0.01*.

**Figure 3:**
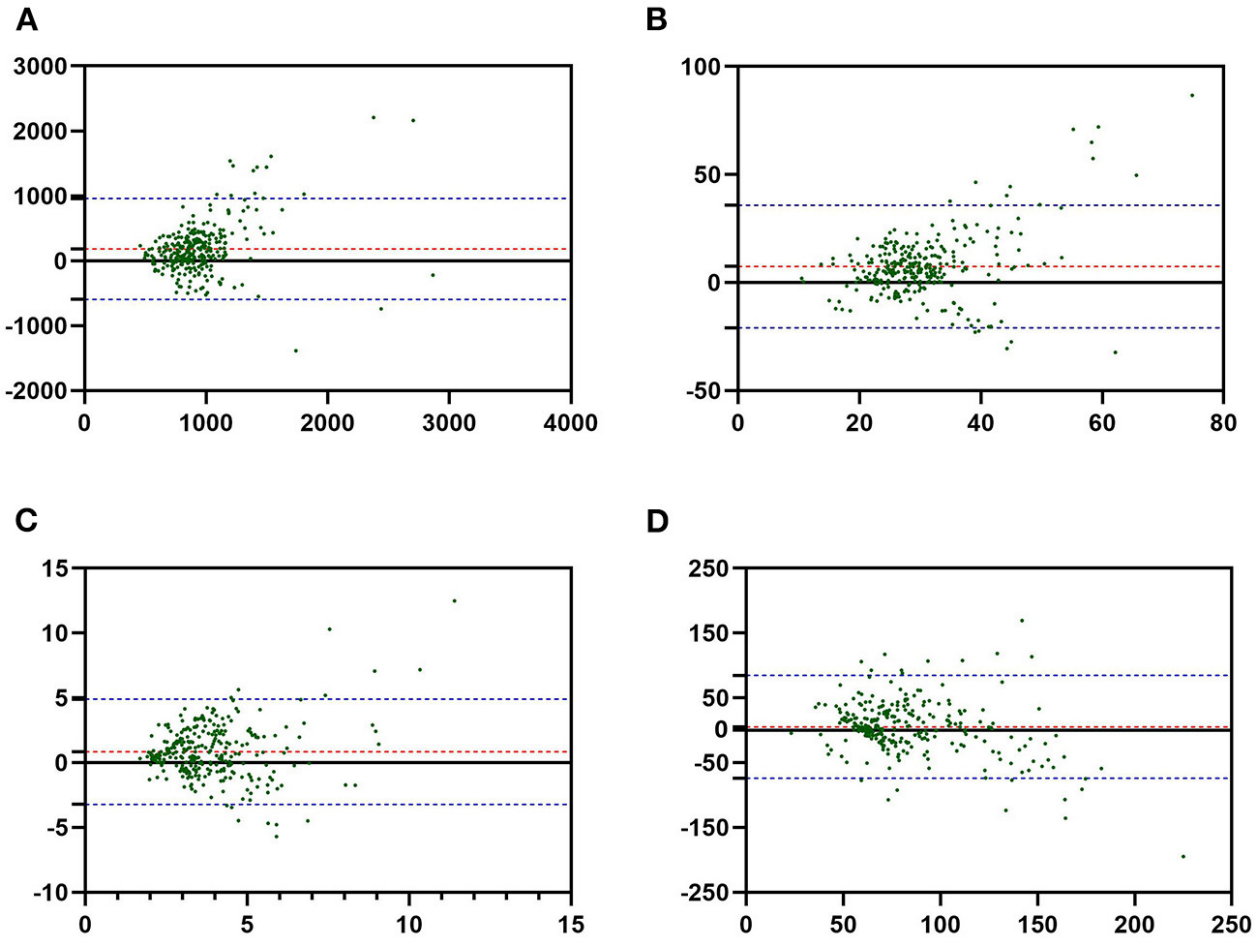
The Bland–Altman plots for energy and select nutrients. **(A)** Energy, **(B)** protein, **(C)** dietary fiber, **(D)** iron. The average intakes from both the food frequency questionnaire 1 (FFQ1) and 3-day 24-h diet recall record (3R24) are plotted on the *X*-axis, and the difference is plotted on the *Y*-axis. The red line means the mean of difference. Two blue lines mean the limits of agreement (LOA), defined as mean difference ± 1.96 × SD. The green points mean dietary intakes data of participants.

The Bland–Altman plots revealed the effective comparability of dietary intake estimated from the FFQ1 and 3R24. The Bland–Altman plots for energy and select nutrients are presented in Figure 3. The mean difference in energy, protein, and dietary fiber values increased with increasing average intakes and most values were within the 95% LOA.

### Dietary Intake Survey

Among the initial 820 participants, 47 were excluded, because of clinical illnesses (*n* = 15), nutritional disease (*n* = 20), and younger than 18 years old (*n* = 12). Thus, 773 (94.26%) participants completed the FFQs in the nutrition intake survey. Finally, we included 1,071 participants (298 participants included in validation analysis were added to 773) in the assessment of nutrition intake, with a mean age of 32.3 years. The majority of participants aged between 18 and 35, and about 50% participants had normal BMI. About 60% participants had low education either from city or rural, and about 50% male participants were illiterate. More than 50% participants were herdsmen. Over 85% of all participants had never smoked or consumed alcohol, which was an unexpected finding. The characteristics of the study population are described in Supplementary Table 2.

The most consumed foods were Tibetan traditional foods, such as Zanba, yak, Tibetan milk tea, Tibetan salt cream tea, Tibetan sweet tea, and Tibetan noodles. In particular, the consumption frequencies of Tibetan salt cream tea and Zanba were once per day almost. Other traditional foods, such as Tibetan noodles, yak, and Tibetan milk tea, were consumed about 2–4 times per week. In contrast, most of vegetables and fruits were consumed 1–3 times or even less than once per month (Table 2).

**Table 2:** The five most frequently consumed foods from the seven food groups.

**Order of frequency**	**Staple**	**Meat**	**Milk**	**Snacks**	**Drinks**	**Vegetables**	**Fruits**
First	Rice^(7)^	Yak^(6)^	Milk^(6)^	Bread^(4)^	Tibetan salt cream tea^(6)^	Potato^(5)^	Apple^(4)^
Second	Zanba^(6)^	Chicken^(4)^	Tibetan milk tea^(6)^	Suger^(4)^	Tibetan tea^(6)^	Chinese cabbage^(4)^	Orange^(4)^
Third	Tibetan noodles^(5)^	Pork^(4)^	Yogurt^(5)^	Instant noodles^(4)^	Cola^(4)^	Carrot^(3)^	Watermelon^(2)^
Fourth	Corn^(4)^	Mutton^(4)^	Yak milk^(4)^	Biscuits^(4)^	Tibetan sweet tea^(4)^	Green cabbage^(3)^	Banana^(2)^
Fifth	Wheat noodles^(4)^	Sausage^(3)^	^*^	Cake^(3)^	Juice^(3)^	Hot pepper^(2)^	Grape^(2)^

*Categories are: 7 (once per day), 6 (5–6 times per week), 5 (2–4 times per week), 4 (once per week), 3 (1–3 times per month), 2 (less than once per month)*.

* *Other foods consumed less than one time per month*.

Most nutrient intakes were significantly lower than the recommended intakes by the Chinese DRIs (Table 3). The median daily energy, for men, was significantly lower than recommended daily energy intake by Chinese DRIs, and the median daily energy was not significant for women. The protein intake was 38.69 and 41.41 g for men and women, respectively. The protein daily intake was significantly lower than daily protein intake recommended by Chinese DRIs for men and women, respectively (Table 3). As for micronutrients, for male participants, the median daily intake of vitamin B1, vitamin B3, vitamin C, potassium, iron, calcium, magnesium, manganese, zinc, and selenium were lower than RNI/AI, particularly vitamin C. While sodium intake was severely more than RNI/AI (Table 3). For the female participants, sodium intake was extremely higher than RNI/AI. However, the intakes of vitamin C, potassium, and calcium were inadequate.

**Table 3:** Daily nutrient intakes of inhabitants in high altitude compared with recommended nutrient intake (RNI) or adequate intake (AI).

**Nutrient**	**Male**				**Female**		
	**Inhabitants**	**RNI/AI**	**(%)**	***P***	**Inhabitants**	**RNI/AI**	**(%)**	***P***
Energy (kcal/d)	1826	2250	81.16	0.000	1990	2150	92.58	0.132
Protein (g/d)	38.69	65.00	59.52	0.000	41.41	55.00	75.29	0.000
Fat (g/d)	137.64	-	-		151.02	-	-	
Carbohydrate (g/d)	121.92	-	-		127.71	-	-	
Dietary fiber (g/d)	5.41	-	-		5.64	-	-	
Vitamin B1(mg/d)	0.47	1.4	33.57	0.000	0.50	1.2	41.67	0.000
Vitamin B3(mg/d)	7.33	12	61.08	0.000	7.79	15	51.93	0.000
Vitamin C (mg/d)	25.35	100.00	25.35	0.000	26.52	100.00	26.52	0.000
Potassium (mg/d)	700.94	2000	35.05	0.000	779.47	2000	38.97	0.000
Sodium (mg/d)	3003.46	1500.00	200.23	0.000	3074.82	1500.00	204.93	0.000
Iron (mg/d)	10.93	12.00	91.08	0.464	11.86	20.00	59.30	0.000
Calcium (mg/d)	428.67	800.00	53.58	0.000	506.41	800.00	63.30	0.000
Magnesium (mg/d)	136.41	330	41.34	0.000	147.88	330	44.81	0.000
Manganese (mg/d)	2.58	4.5	57.33	0.000	2.66	4.5	59.11	0.000
Zinc (mg/d)	4.93	12.5	39.44	0.000	5.15	7.5	68.67	0.000
Selenium (ug/d)	24.51	60	40.85	0.000	26.55	60	44.25	0.000

*(%) = nutrient intakes of inhabitants were divided by RNI or AI*.

Most dietary intakes of Tibetans were significantly different from the data from the 2011CHNS, either male or female. Our analysis used relevant data from the 2011CHNS. According to the inclusion and exclusion criteria, 4,890 participants were included in our study finally. The intake of energy was similar to the Shandong province, but the intakes of energy, fat, vitamin A, and sodium were significantly higher than those reported in the 2011CHNS. Intakes of other nutrients were significantly lower than that in the 2011CHNS. Similar findings could be observed in other provinces (Table 4). According to Table 5, analysis of dietary intakes based on sex showed that participants in high-altitude areas had significantly lower intakes of protein, carbohydrate, dietary fiber, cholesterol, vitamin C, calcium, and iron than those reported in the 2011CHNS. Intakes of other nutrients were significantly higher than in the 2011CHNS, especially sodium. There were no sex-based differences in these results.

**Table 4:** Daily nutrient intakes of inhabitants in high altitude compared with 2011 China Health and Nutrition Survey (2011CHNS).

**Nutrient**	**Inhabitants (1071)**	**2011CHNS**						
		**All (4890)**	**Beijing (982)**	**Liaoning (774)**	**Heilongjiang (829)**	**Shanghai (1075)**	**Jiangsu (834)**	**Shandong (396)**
Energy (kcal/d)	1934	1538^**^	1393^**^	1675^**^	1837^**^	1299^**^	1658^**^	1979
Protein (g/d)	39.95	56.14^**^	49.49^**^	54.60^**^	56.06^**^	54.66^**^	63.30^**^	64.06^**^
Fat (g/d)	146.22	35.6^**^	33.52^**^	30.65^**^	30.22^**^	40.48^**^	38.83^**^	44.54^**^
Carbohydrate (g/d)	124.45	221.36^**^	194.60^**^	250.61^**^	278.29^**^	167.49^**^	255.92^**^	275.78^**^
Dietary fiber (g/d)	5.52	23.19^**^	27.36^**^	28.08^**^	22.95^**^	16.86^**^	16.33^**^	44.03^**^
Cholesterol (mg/d)	122.14	246.24^**^	282.44^**^	201.43^**^	201.16^**^	264.52^**^	232.40^**^	328.80^*^
Vitamin A (mg/d)	799.95	154.86^**^	214.59^**^	108.08^**^	116.30^**^	170.63^**^	155.37^**^	159.97^**^
Vitamin C (mg/d)	25.96	71.3^**^	95.94^**^	71.35^**^	95.16^**^	46.54^**^	58.99^**^	83.91^**^
Calcium (mg/d)	460.61	768.4^**^	705.90^**^	794.07^**^	831.68^**^	688.18^**^	816.70^*^	906.32^**^
Sodium (mg/d)	3030.18	556.2^**^	572.21^**^	457.77^**^	488.13^**^	532.91^**^	548.87^**^	829.50^**^
Iron (mg/d)	11.45	15.33^**^	13.38^**^	14.71^**^	19.53^**^	14.69^**^	14.19^**^	19.12^**^

* *p < 0.05*,

** *p < 0.01*.

**Table 5:** Daily nutrient intakes of inhabitants in high altitude compared with 2011CHNS by sex.

**Nutrient**	**Male (2852)**		**Female (3109)**	
	**Tibetan inhabitants (557)**	**2011CHNS (2295)**	**Tibetan inhabitants (514)**	**2011CHNS (2595)**
Energy (kcal/d)	1826	1696^*^	1990	1425^**^
Protein (g/d)	38.69	61.45^**^	41.41	50.98^**^
Fat (g/d)	137.64	39.47^**^	151.02	32.76^**^
Carbohydrate (g/d)	121.92	243.54^**^	127.71	203.58^**^
Dietary fiber (g/d)	5.41	24.50^**^	5.64	22.16^**^
Cholesterol (mg/d)	113.21	263.34^**^	135.7	230.25^**^
Vitamin A (mg/d)	747.32	158.94^**^	863.64	150.65^**^
Vitamin C (mg/d)	25.35	73.03^**^	26.52	69.47^**^
Vitamin E (mg/d)	93.77	10.39^**^	107.69	9.15^**^
Calcium (mg/d)	428.67	831.04^**^	506.41	719.86^**^
Sodium (mg/d)	3003.46	604.71^**^	3074.82	509.98^**^
Iron (mg/d)	10.93	16.67^**^	11.86	14.21^**^

* *p < 0.05*,

** *p < 0.01*.

## Discussion

### Validity and Reproducibility of FFQ

In this study, we reported the relative validity and reproducibility of a 125-food item FFQ designed for inhabitants in Tibet. According to the results of validation and reproducibility, it showed that the Tibetan version FFQ is acceptable valid and reproducible in assessing the food groups and nutrients intakes.

In the analysis of validation, a major component was the appropriate reference method. In a review on the validation of FFQs, the authors showed that majority of the studies assessed the FFQs and 3R24s (27). The correlation coefficients that ranged from 0.45 to 0.70 were evident among validation studies that used questionnaires (28). A study in a large Chinese population showed that the correlation coefficients of all food groups after energy adjustment varied from 0.28 to 0.63, with correlations ranging from 0.37 to 0.98 for all nutrients (29). In our study, relative validity was tested by comparing FFQ1 with the average of 3R24 as well. The Pearson's correlation values (adjusted) of food group intakes varied from 0.21 to 0.99. The correlation coefficients of nutrients (adjusted) ranged from 0.23 to 0.75, with most values above 0.30. Several validation studies reported similar correlation coefficients between the FFQ and 3R24 to ours (30, 31). Comparing with previous studies, our results indicated acceptable correlation in our Tibetan version FFQ. The included population was a group of inhabitants in Tibet. The majority of inhabitants failed to access higher education and were not capable of understanding the abstract concepts. These factors could influence the assessment of validity. Upon conducting a reproducibility evaluation, we analyzed the agreement of FFQ1 and FFQ2. When a long interval of time was used, true changes in dietary intake and differences in response, may lead to the reduction of reproducibility (18). In this study, we used 2 months as the interval, which we believe was suitable. A moderate degree of reproducibility was shown for both food consumption and nutrient intake, with the ICC (adjusted) ranging from 0.24 to 0.68 and varying from 0.22 to 0.84, respectively. The FFQ developed for women in China showed that the ICC ranged from 0.45 to 0.86 (32). The time interval in the study was 4 weeks shorter than that in our study. According to a meta-analysis about the reproducibility of FFQ, the FFQs with correlation coefficients >0.5 for most nutrients may be considered reliable tools to measure dietary intake (33).

### Dietary Intake Survey

Our dietary survey provided detailed information on the daily nutrient intakes of Tibetan inhabitants. According to the analysis of data of 1,071 participants, we assessed daily nutrition intake of high-altitude inhabitants and compared our results with Chinese DRIs and the 2011CHNS data.

The most demographic characteristics of participants included our research were similar to the people living in high altitude areas rather than low altitude areas. The people living in high altitude areas tend to be leaner than those living in low altitude areas (34). In addition, it has been established that obesity is not common in high altitude areas (34–36). Zhao et al. found that the prevalence of overweight and obesity (defined as BMI ≥ 25 and 30 kg/m^2^) among Tibet minorities in China ranged from 6.3% to 18.4% (37). The prevalence of obesity among adult living at an altitude of 3,660 m was 9.7% (38). Our analysis showed the rates of overweight and obesity were 23.34% and 11.30%, respectively, differed somewhat from previous studies (37, 38). The rate of overweight was slightly higher than 18.4%, which could be due to decreased physical activity level at high altitude. However, the rate of obesity was similar to 9.7% and 18.4%. The other demographic features of our participants, such as education level and smoking and drinking status, were generally in consistent with those of people in other studies (38, 39).

Compared with Han Chinese, the dominant population of China, dietary pattern of Tibetans was considerably distinct. Approximately 25% of foods consumed frequently were traditional Tibetan foods, such as Zanba, yak, Tibetan salt cream tea. In addition, the consumptions of vegetables and fruits were less frequent than Han Chinese, which were also reported by other researchers (40). Similar dietary patterns have been observed among other minority nationalities in China (41). Compared with previous findings (42), our analysis showed Tibetans consumed less energy and protein compared with their counterparts in flat areas in China. The difference in dietary intake might be attributed to various characteristics specific to Tibet, such as the vast area, inconvenient traffic, long winter, low temperature influenced by cold, and low oxygen environments.

Fat and sodium intakes were significantly higher than recommended daily intake by Chinese DRIs. We found that energy and iron or protein intakes, for men and women respectively, were close to the Chinese DRIs. However, fat and sodium intakes were significantly higher than the Chinese DRIs. Over consumption of animal-based foods, and/or especially oil may contribute to high fat intake. According to a previous study, the incidence of various liver diseases, such as fat liver was 29.89% among 696 Tibetan people (43). This might be attributed to over intake of fat. In addition, studies conducted in other Chinese populations (44, 45) suggested that high sodium consumption remained a public health problem. However, the problem can be more serious in Tibet inhabitants. In the study, significantly higher sodium levels were found among the Tibetan population compared with others in China (46). Therefore, control of sodium intake should be a significant measure. Furthermore, we determined that other nutrients, especially vitamin C, showed low intake extremely. A cross-sectional survey that was conducted to analyze the nutrient intakes of rural Tibetan mothers (16) showed insufficient intake of vitamin C. In our study, the main reason may be the reduced frequency of consumption of fresh vegetables and fruits. These foods accounted for more than 35 items in our FFQ, but only five items had a frequency of at least once per week; other foods in these subgroups were consumed less frequently, which indicated that it is necessary to increase the fresh vegetables and fruits intakes for Tibetan inhabitants.

**Figure 4:**
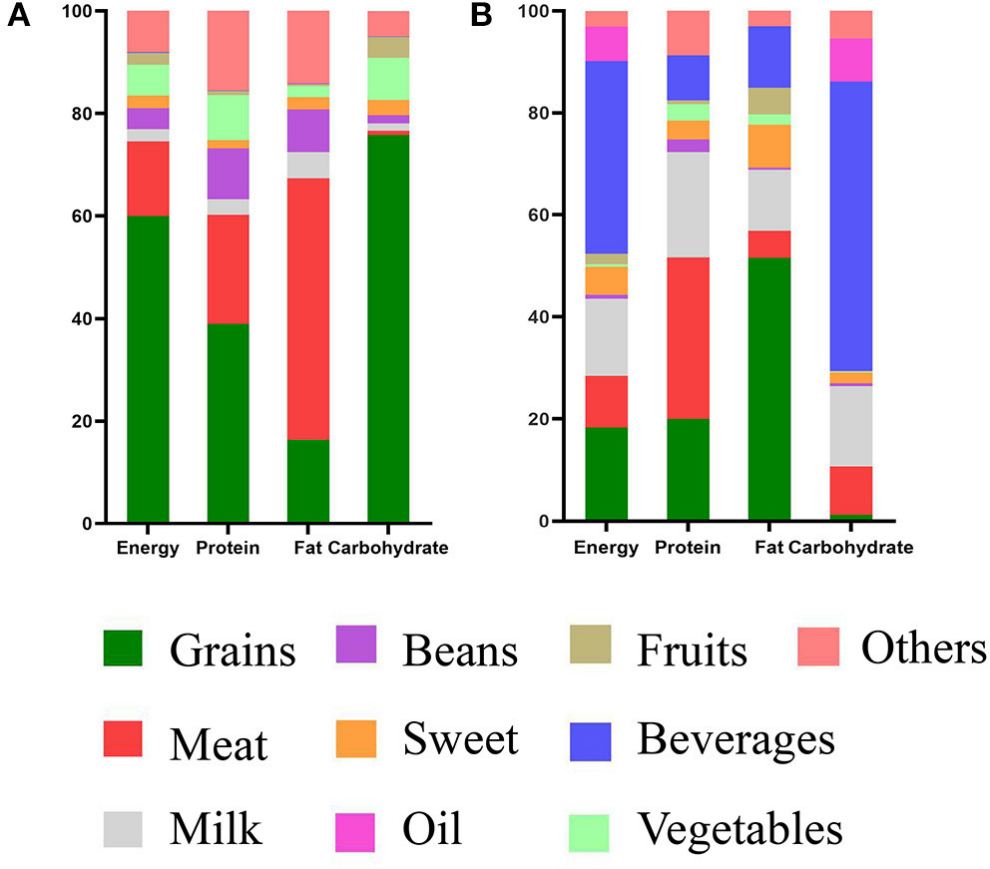
Food sources of energy and macronutrients among inhabitants in high altitude and 2011CHNS. **(A)** Nutrient intakes of 2011CHNS, **(B)** nutrient intakes of inhabitants. The nutrition of participants consumed are plotted on the *X*-axis, and percentage contribution of the nine major food groups are plotted on the *Y*-axis.

Comparing with the 2011CHNS data, energy and macronutrient (fat, protein, and carbohydrate) consumption showed significant differences. Figure 4 depicts the percentage contribution of the nine major food groups to energy and macronutrients. The contributions of the major food groups were increasingly different in the two populations. The top three major food sources of energy were beverages (37.78%), grains (18.30), and milk (15.25%) among high-altitude inhabitants; However, the food sources were different in the 2011CHNS data—the main food source for energy was grains (60.02%). This was likely because of over consumption of Tibetan salt cream tea in Tibet. Local inhabitants consumed Tibetan salt cream tea almost once per day. Similar results were observed for carbohydrate intake. In data from Tibetan inhabitants and the 2011CHNS, the main food group was shown to be beverages and grain, respectively. For high-quality protein intake, Tibetan inhabitants consumed high levels of meat and milk instead of grain, so that the prevalence of protein deficiency diseases might be low because of consumption high-quality protein. In terms of fat intake, the main food source was grain, which was likely attributed to the high intake of Zanba. Overall, Tibetan inhabitants followed both traditional (grain and meat) and special (high fat and sodium) dietary patterns.

The strengths of our study are that we developed an appropriate FFQ that included 125 food items typically consumed by Tibetan inhabitants. According to our study, the FFQ can be used to appropriately evaluate the dietary intake of inhabitants. Furthermore, the validity of the FFQ was evaluated with not only correlation coefficients but also the Bland–Altman method that has been preferred over correlation analysis. Finally, in the dietary survey, we included 1,071 inhabitants. Our result has provided a comprehensive summary of the dietary intakes and the base to assessment nutritional status among Tibetan inhabitants. Our study has some limitations. First, the food groups designed did not include fish, which may influence protein and other nutrient intakes. Second, the length of the FFQ could have influenced participant responses, given that other shorter FFQs have been used previously. This may also have contributed to the overestimation of food and nutrient intakes in this study. Last, the sample size of the surveyed population was relatively small, because we only collected data from inhabitants of the North of Tibet. Thus, the study lacks generalizability. Further research is warranted to assess the current dietary intakes trends and features among high-altitude populations.

## Conclusions

We developed a 125-food item FFQ with good validity and reproducibility to assess the dietary intakes among inhabitants of the North of Tibet. The results of nutritional analysis showed that ~25% of foods consumed frequently were traditional Tibetan foods. However, traditional Han foods were frequently consumed as well. In addition, over consumption of fat and sodium and low intakes of other nutrients, especially vitamin C, were the most significant findings. Hence, it is necessary to control animal-based foods and/or oil consumption in particular and increase the consumption of fresh vegetables and fruits.

## Data Availability Statement

The raw data supporting the conclusions of this article will be made available by the authors, without undue reservation.

## Ethics Statement

The studies involving human participants were reviewed and approved by Naqu People's Hospital. The patients/participants provided their written informed consent to participate in this study.

## Author Contributions

XS: original draft preparation, methodology, software analysis, formal analysis, and data curation. ZX: investigation, resources, manuscript review, and editing. XL, XX, and ZX: supervision and project administration. All authors have read and agreed to version submitted for publication.

## Funding

This study was supported by the Tibet Local Science and Technology Project guided by the Central Government (Grant No. XZ202001YD0005C), Scientifical Funds of Medical Assistance Program for Tibet from the Tibet Health Committee [Grant Nos. XZ2020ZR-ZY77(Z), XZ2020ZRZY78(Z)], Natural Science Funds of Liaoning (Grant No. 2019-BS-073), and Scientific technology project of Liaoning Education Administration (Grant No. LZ2019044).

## Conflict of Interest

The authors declare that the research was conducted in the absence of any commercial or financial relationships that could be construed as a potential conflict of interest.

## Publisher's Note

All claims expressed in this article are solely those of the authors and do not necessarily represent those of their affiliated organizations, or those of the publisher, the editors and the reviewers. Any product that may be evaluated in this article, or claim that may be made by its manufacturer, is not guaranteed or endorsed by the publisher.
