# Supplementary material for: Distinct Nutrient Intake Style in Inhabitants of Ultra-High-Altitude Areas in North of Tibet, China: A Cross-Sectional Study Based on Newly Developed Tibetan Food Frequency Questionnaires
Source: Front Nutr. 2021 Dec 23;8:743896. doi: 10.3389/fnut.2021.743896 (PMC8733569; doi:10.3389/fnut.2021.743896)
Supplement: Supplementary file 1 [file Table_1.DOCX]

Supplementary Table 1. The characteristics of the participants in the validity study.

| Baseline Characteristic | City (N=99) | | Rural (N=199) | | Total (N=298) |
| --- | --- | --- | --- | --- | --- |
|  | Male(N=49) | Female(N=50) | Male(N=95) | Female(N=104) |  |
| Age, mean (SD) | 36.92(12.02) | 30.40(10.76) | 35.27(12.43) | 34.42(11.31) | 32.23(12.08) |
| BMI, n (%) |  |  |  |  |  |
| <18.5 | 6(12.24) | 17(34.00) | 14(14.74) | 25(24.04) | 62(20.81) |
| 18.5–24.9 | 34(69.39) | 26(52.00) | 62(65.26) | 65(62.50) | 187(62.75) |
| 25–29.9 | 4(8.17) | 4(8.00) | 14(14.74) | 7(6.73) | 29(9.73) |
| >30 | 5(10.20) | 3(6.00) | 5(5.26) | 7(6.73) | 20(6.71) |
| Education (n%) |  |  |  |  |  |
| Illiterate | 28(57.14) | 25(50.00) | 56(58.95) | 63(60.58) | 172(57.72) |
| Low | 6(12.24) | 4(8.00) | 9(9.47) | 9(8.65) | 28(9.39) |
| Medium | 7(14.28) | 5(10.00) | 18(18.95) | 18(17.31) | 48(16.11) |
| High | 8(16.34) | 16(32.00) | 12(12.63) | 14(13.46) | 50(16.78) |
| Monthly Income (¥) (n%) |  |  |  |  |  |
| <2000 | 21(42.85) | 19(38.00) | 46(48.43) | 41(39.42) | 127(42.62) |
| 2000–5000 | 8(16.33) | 9(18.00) | 15 (15.79) | 23(22.12) | 55(18.46) |
| 5000–8000 | 12(24.49) | 14(28.00) | 17(17.89) | 23(22.12) | 66(22.15) |
| >8000 | 8(16.33) | 8(16.00) | 17(17.89) | 17(16.34) | 50(16.77) |
| Marital Status (n%) |  |  |  |  |  |
| Married | 39(79.59) | 31(62.00) | 77(81.05) | 87(83.65) | 234(78.52) |
| Never Married | 10(20.41) | 18(36.00) | 16(16.84) | 17(16.35) | 61(20.47) |
| Others | 0 | 1(2.00) | 2(2.11) | 0 | 3(1.00) |
| Employment Status (n%) |  |  |  |  |  |
| Not Employed | 2(4.08) | 4(8.00) | 88.42 | 8(7.69) | 22(7.38) |
| Herdsman | 33(67.34) | 29(58.00) | 65(68.42) | 78(75.00) | 205(68.79) |
| Worker | 1(2.05) | 2(4.00) | 4(4.21) | 4(3.85) | 11(3.69) |
| Others | 13(26.53) | 15(30.00) | 18(18.95) | 14(13.46) | 60(20.14) |
| Smoking (n%) |  |  |  |  |  |
| Never | 37(75.51) | 49(98.00) | 74(77.89) | 92(88.46) | 252(84.56) |
| Former | 2(4.08) | 0 | 2(2.11) | 1(0.96) | 5(1.68) |
| Current | 10(20.41) | 1(2.00) | 19(20.00) | 11(10.58) | 41(13.76) |
| Drinking (n%) |  |  |  |  |  |
| Never | 37(75.51) | 46(92.00) | 81(85.26) | 92(88.46) | 256(85.91) |
| Former | 3(6.12) | 2(4.00) | 2(2.11) | 4(3.85) | 11(3.69) |
| Current | 9(18.37) | 2(4.00) | 12(12.63) | 8(7.69) | 31(10.40) |

Supplementary Table 2. The characteristics of the participants in the dietary intake survey.

| Baseline Characteristic | City (N=347) | | Rural (N=724) | | Total (N=1071) |
| --- | --- | --- | --- | --- | --- |
|  | Male (188) | Female (159) | Male (369) | Female (355) |  |
| Age, n (%) |  |  |  |  |  |
| 18-35 | 106(56.38) | 118(74.21) | 228(61.79) | 257(72.39) | 709(66.20) |
| 36-50 | 51(27.13) | 27(16.98) | 103(27.91) | 64(18.03) | 245(22.88) |
| 51-65 | 31(16.49) | 14(8.81) | 38(10.30) | 34(9.58) | 117(10.92) |
| BMI, n (%) |  |  |  |  |  |
| <18.5 | 11(5.85) | 31(19.50) | 33(8.94) | 59(16.62) | 134(12.51) |
| 18.5–24.9 | 91(48.41) | 82(51.57) | 185(50.13) | 208(58.59) | 566(52.85) |
| 25–29.9 | 58(30.85) | 27(16.98) | 111(30.08) | 54(15.21) | 250(23.34) |
| >30 | 28(14.89) | 19(11.95) | 40(10.85) | 34(9.58) | 121(11.30) |
| Education (n%) |  |  |  |  |  |
| Illiterate | 97(51.60) | 73(45.91) | 188(50.94) | 147(41.41) | 505(47.15) |
| Low | 27(14.36) | 19(1.95) | 36(9.76) | 52(14.65) | 134(12.51) |
| Medium | 35(18.62) | 37(23.27) | 80(21.68) | 100(28.17) | 252(23.53) |
| High | 29(15.42) | 30(18.87) | 65(17.62) | 56(15.77) | 180(16.81) |
| Monthly Income (¥) (n%) |  |  |  |  |  |
| <2000 | 57(30.32) | 56(35.22) | 133(36.04) | 100(28.17) | 346(32.31) |
| 2000–5000 | 71(37.77) | 49(30.82) | 109(29.54) | 146(41.13) | 375(35.01) |
| 5000–8000 | 42(22.34) | 34(21.38) | 76(20.60) | 70(19.72) | 222(20.73) |
| >8000–11000 | 18(9.57) | 20(12.58) | 51(13.82) | 39(10.98) | 128(11.95) |
| Marital Status (n%) |  |  |  |  |  |
| Married | 125(66.49) | 90(56.61) | 250(67.75) | 195(54.93) | 660(61.63) |
| Never Married | 62(32.98) | 61(38.36) | 114(30.89) | 156(43.94) | 393(36.69) |
| Others | 1(0.53) | 8(5.03) | 5(1.36) | 4(1.13) | 18(1.68) |
| Employment Status (n%) |  |  |  |  |  |
| Not Employed | 5(2.66) | 8(5.03) | 18(4.88) | 10(2.82) | 41(3.83) |
| herdsman | 109(57.98) | 81(50.94) | 222(60.17) | 250(70.42) | 662(61.81) |
| worker | 23(12.23) | 23(14.47) | 40(10.84) | 30(8.45) | 116(10.83) |
| others | 51(27.13) | 47(29.56) | 89(24.11) | 65(18.31) | 252(23.53) |
| Smoking (n%) |  |  |  |  |  |
| Never | 172(91.48) | 141(88.69) | 306(82.93) | 306(86.20) | 925(86.37) |
| Former | 8(4.26) | 6(3.77) | 16(4.34) | 9(2.54) | 39(3.64) |
| Current | 8(4.26) | 12(7.54) | 47(12.73) | 40(11.26) | 107(9.99) |
| Drinking (n%) |  |  |  |  |  |
| Never | 160(85.1) | 138(86.79) | 321(86.99) | 320(90.14) | 939(87.67) |
| Former | 15(7.98） | 4(2.52) | 10(2.71) | 6(1.69) | 35(3.27) |
| Current | 13(6.91) | 17(10.69) | 38(10.30) | 29(8.17) | 97(9.06) |
